# Branch Retinal Vein Occlusion in a COVID-19 Positive Patient

**DOI:** 10.7759/cureus.13586

**Published:** 2021-02-27

**Authors:** Sarah Madison Duff, Matthew Wilde, Gibran Khurshid

**Affiliations:** 1 Ophthalmology, University of Florida, Gainesville, USA

**Keywords:** branch retinal vein occlusion, retinal vein occlusion, covid-19, sars-cov-2, severe acute respiratory syndrome, thromboembolic events

## Abstract

Retinal vein occlusions (RVOs), including central retinal vein occlusions (CRVOs) and branch retinal vein occlusions (BRVOs), are a common cause of morbidity in elderly patients. We present the case of a healthy 74-year-old female patient who initially presented with blurry vision in her left eye in the setting of a symptomatic COVID-19 infection. She was diagnosed with a branch retinal vein occlusion that did not immediately require treatment. Three months later, she again presented with worsening vision and was found to have cystoid macular edema (CME) secondary to the vein occlusion, thus was treated with an intravitreal dexamethasone implant. This case serves to highlight the growing evidence of increased thromboembolic risk associated with severe acute respiratory syndrome coronavirus 2 (SARS-CoV-2) and the possible correlation of COVID-19 infections with ocular pathology, including retinal vein occlusions.

## Introduction

Despite the mounting collection of scientific research on severe acute respiratory syndrome coronavirus 2 (SARS-CoV-2), the virus responsible for the COVID-19 pandemic, the pathogenesis of this severe acute respiratory syndrome (SARS) virus continues to be poorly understood. Among those treating COVID-19 patients, the SARS-CoV-2-induced cytokine storm is a recognized cause of morbidity and mortality and a growing collection of data supports COVID-19 predisposing patients to thromboembolic events with a multitude of systemic adverse effects, including pulmonary emboli and deep vein thromboses [1,2].

In the realm of ocular disease, retinal findings such as cotton wool spots and retinal hemorrhages have been found in otherwise healthy COVID-19 positive patients [3], leading one to speculate that the retina may not be protected from these thromboembolic events. As the pandemic has continued, reports of possible retinal thrombotic complications in patients diagnosed with COVID-19 have continued to surface [4,5].

Retinal vein occlusions (RVOs) are a widespread cause of morbidity in elderly patients and are known to be associated with thromboembolic events. We report a 74-year-old individual diagnosed with a branch retinal vein occlusion (BRVO) while symptomatic with COVID-19.

## Case presentation

A 74-year-old, Caucasian female with a past medical history of well-controlled hyperlipidemia presented to the emergency department with worsening vision of the left eye. Approximately three months prior, the patient had been diagnosed with SARS-CoV-2. While symptomatic from COVID-19, she developed blurry vision in the left eye and was diagnosed with a branch retinal vein occlusion (BRVO) that did not require treatment at that time. The exact time of COVID-19 onset to retinal vascular event is not known, however, our patient was symptomatic with COVID-19 when she became symptomatic from a change in her vision in her left eye.

**Figure 1:**
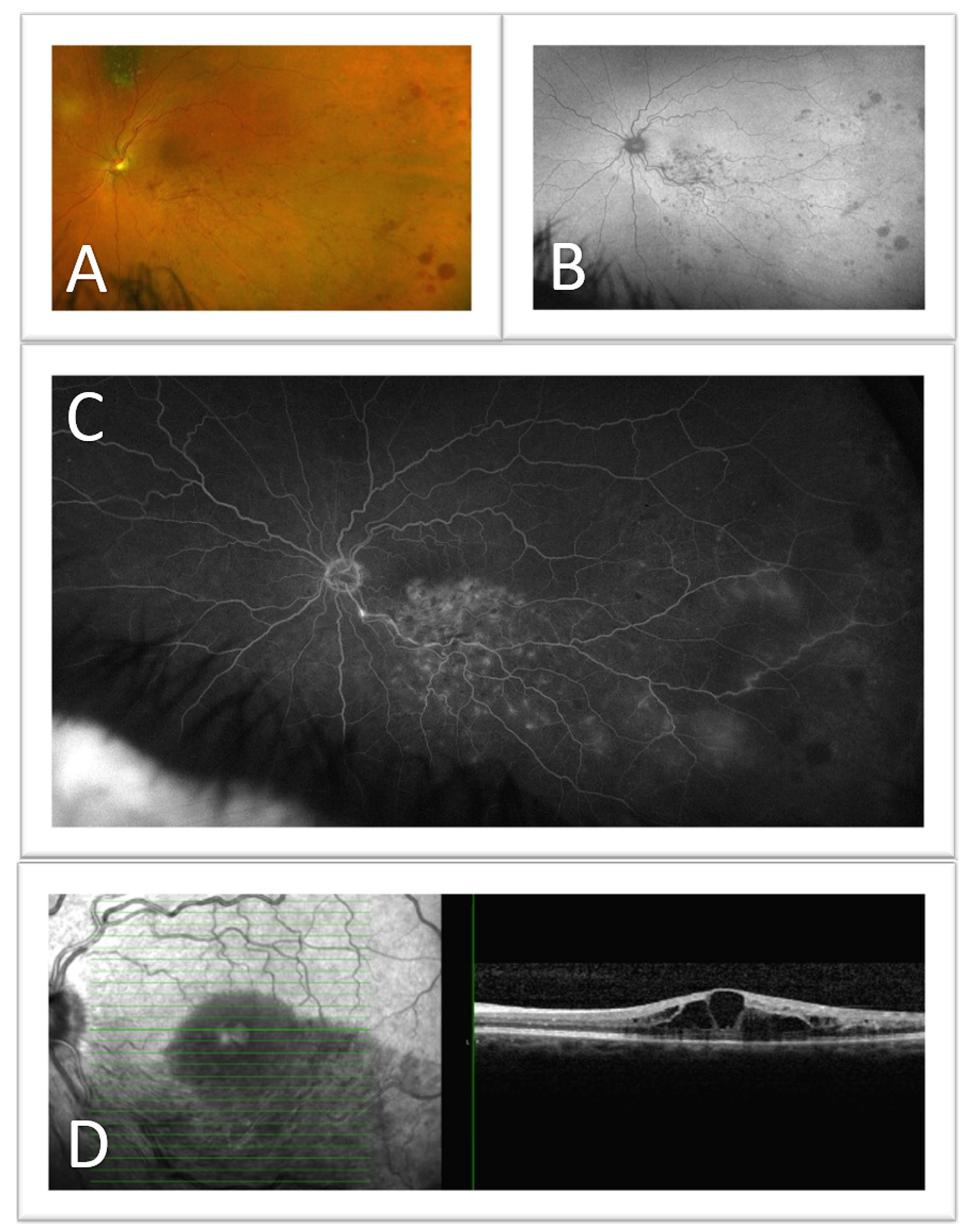
Cystoid macular edema (CME) in the setting of a branch retinal vein occlusion (BRVO) of the left eye (A) Color fundus photo, (B) Fundus autofluorescence (FAF), (C) Fluorescein angiography, and (D) optical coherence tomography (OCT) show the left eye of a 74-year-old female with a choroidal nevus superior to the optic nerve, retinal hemorrhaging inferotemporally along the inferior retinal vein, and macular edema.

On this examination three months later, the vision in the right eye was 20/25, while the vision in the left eye was best corrected to 20/50 at distance. Intraocular pressures were normal at 11 and 12 millimeters of mercury (mmHg) in the right and left eyes, respectively, measured with a TonoPen tonometer. Anterior examination was within normal limits for age, with nuclear sclerosis present in both eyes. Dilated fundus examination showed a choroidal nevus peripherally in the right eye. In the left eye, tortuous vessels with intraretinal hemorrhages and microaneurysms were present in the inferior-temporal quadrant of the retina with macular edema centrally, in addition to a peripheral choroidal nevus with overlying drusen and without surrounding subretinal fluid. Imaging was obtained for further evaluation and a diagnosis of BRVO with cystoid macular edema (CME) of the left eye was confirmed (Figure 1).

Based on the new-onset macular edema in the setting of a BRVO, the patient was treated with intravitreal dexamethasone implant with improvement of visual symptoms and resolution of intraretinal fluid on optical coherence tomography (OCT). Alternatively, intravitreal anti-vascular endothelial growth factor (Anti-VEGF) therapy could have been implemented. She continued to follow closely with her primary care physician to ensure that her hyperlipidemia continued to be well controlled, as well as be monitored for blood pressure and glucose control. A further hypercoagulability work-up was not done. Due to the BRVO being symptomatic three months after its initial presentation and the patient no longer being symptomatic from the SARS-CoV-2 virus, systemic treatment, such as an anticoagulant, was not initiated.

## Discussion

Retinal vein occlusions (RVOs) are a common cause of morbidity in elderly patients. RVOs include central retinal vein occlusions (CRVOs) and branch retinal vein occlusions (BRVOs) and may cause blurry vision or severe loss of vision, depending on the location and severity of obstruction. Causes of visual decline in BRVOs include macular edema, ischemia, neovascularization, and hemorrhage. A multitude of risk factors have been indicated in RVOs including atherosclerotic disease, hypertension, diabetes mellitus, open angle glaucoma, connective tissue diseases, thrombophilia, and other causes of hypercoagulability. In our patient, her development of a BRVO of the left eye coincided with her diagnosis and symptoms of COVID-19.

Coronaviruses (CoVs) affecting ocular tissue is not a novel discovery. Seven of the CoVs currently infect humans and, while most cause respiratory tract infections, three of the seven have also been detected in ocular tissue or shown to cause ocular symptoms, including the current SARS-CoV-2 virus [6]. HCoV-NL63 was identified in 2004, isolated from a seven-month-old child with bronchiolitis and conjunctivitis [7]. Further studies were performed and three of 18 patients determined to have HCoV-NL63 also presented with conjunctivitis [8]. SARS-CoV, the virus responsible for severe acute respiratory syndrome (SARS) that in 2003 caused a global outbreak, has been detected in tear samples of infected individuals [9]. Additionally, a coronavirus that affects domestic and wild cats rather than humans, feline CoV (FCoV) may manifest as conjunctivitis, anterior uveitis, choroiditis, or retinal vasculitis [10]. Thus, coronaviruses have previously been reported to have ocular involvement, despite limited studies on this topic.

While reasonable to consider a retinal vasculitis, such as that induced by the feline CoV, causing the retinal findings in our patient, rather than a thromboembolic event that we propose, other findings of a retinal vasculitis were not evident at three months after the initial BRAO on the fluorescein angiography. However, a retinal vasculitis rather than a thrombotic event should still be considered on a differential for a COVID-19 patient with ocular changes.

Ribonucleic acid (RNA) from the SARS-CoV-2 virus has been detected in many diverse ocular specimens, including in the tears and conjunctival secretions of patients with coronavirus conjunctivitis [11]. Furthermore, COVID-19 is known to use the angiotensin-converting enzyme 2 (ACE2) receptor, which is expressed in the retina, to gain entry into human cells [12]. Thus, somewhat unsurprisingly, SARS-CoV-2 has been reported, presented by reverse transcriptase-polymerase chain reaction (RT-PCR), in retina samples of deceased patients with confirmed COVID-19 disease [13].

Recent publications have recognized retinal hemorrhages and cotton wool spots in COVID-19 positive patients without other retinal disease [3]. Several reports have recently been published of otherwise healthy individuals presenting with RVO coinciding with their diagnosis of COVID-19. Yahalomi et al. described a healthy 33-year-old suspected COVID-19 patient who presented with a unilateral central retinal vein occlusion [14]. Sheth et al. presented the case of a 52-year-old male diagnosed with COVID-19 who was determined to have a new unilateral vasculitic retinal vein occlusion [15]. Walinjkar er al. reported a 17-year-old girl found to have a CRVO of the right eye also correlating with her diagnosis of COVID-19 [16]. Gaba et al. presented a case of bilateral CRVOs associated with deep vein thrombosis, right heart strain, and hypertension in a 40-year-old male with a severe COVID-19 pneumonia requiring hospital admission [17]. Finally, Invernizzi et al. reported a case of a 54-year-old female with COVID-19 pneumonia treated with systemic steroids for a symptomatic impending central retinal vein occlusion of her right eye [18].

This is the first reported case, to our knowledge, of a branch retinal vein occlusion coinciding with a diagnosis of COVID-19.

## Conclusions

In this case report, we present a branch retinal vein occlusion occurring in association with the diagnosis of COVID-19. Admittedly, the relationship between COVID-19 and RVOs has yet to be completely elucidated. It is fully possible that due to the high numbers of COVID-19 cases worldwide at this time, the simultaneous diagnoses of COVID-19 and retinal vein occlusions is coincidental, however, with the association of COVID-19 and thromboembolic phenomenon, there is also reason to suspect that this is more than a coincidence. Further studies should be performed to better understand the correlation between COVID-19 and retinal pathology, including retinal vein occlusions.
